# Comparative Effect of Antioxidant and Antibacterial Potential of Zinc Oxide Nanoparticles from Aqueous Extract of *Nepeta nepetella* through Different Precursor Concentrations

**DOI:** 10.3390/ma17122853

**Published:** 2024-06-11

**Authors:** Nouzha Fodil, Djaaboub Serra, Johar Amin Ahmed Abdullah, Juan Domínguez-Robles, Alberto Romero, Amrouche Abdelilah

**Affiliations:** 1 Laboratory for Sustainable Management of Natural Resources in Arid and Semi-Arid Areas, University Center of Salhi Ahmed, P.O. Box 66, Naâma 45000, Algeria; amrouche@cuniv-naama.dz; 2 Laboratory of the Valorization of Plant Resources and Food Security in Semi-Arid Areas of Southwest Algeria, Bechar 08000, Algeria; djaaboub.serra@univ-bechar.dz; 3 Department of Chemical Engineering, Faculty of Chemistry, University of Seville, 41012 Seville, Spain; jabdullah@us.es (J.A.A.A.); alromero@us.es (A.R.); 4 Department of Pharmacy and Pharmaceutical Technology, Faculty of Pharmacy, University of Seville, 41012 Seville, Spain

**Keywords:** zinc oxide nanoparticles, ZnO, antibacterial activity, antioxidant activity, green synthesis, *Nepeta nepetella* subps. *amethystine*

## Abstract

Antibiotic resistance is a global health crisis caused by the overuse and misuse of antibiotics. Accordingly, bacteria have developed mechanisms to resist antibiotics. This crisis endangers public health systems and medical procedures, underscoring the urgent need for novel antimicrobial agents. This study focuses on the green synthesis of ZnO nanoparticles (NPs) using aqueous extracts from *Nepeta nepetella* subps. *amethystine* leaves and stems, employing different zinc sulfate concentrations (0.5, 1, and 2 M). NP characterization included transmission electron microscopy (TEM), scanning electron microscopy (SEM), and X-ray diffraction (XRD), along with Fourier transform infrared spectroscopy (FTIR) analysis. This study aimed to assess the efficacy of ZnO NPs, prepared at varying concentrations of zinc sulfate, for their capacity to inhibit both Gram-positive and Gram-negative bacteria, as well as their antioxidant potential using the 2,2-diphenyl-1-picrylhydrazyl (DPPH) method. SEM and TEM results showed predominantly spherical NPs. The smallest size (18.5 ± 1.3 nm for leaves and 18.1 ± 1.3 nm for stems) occurred with the 0.5 M precursor concentration. These NPs also exhibited remarkable antibacterial activity against both Gram-positive and Gram-negative bacteria at 10 µg/mL, as well as the highest antioxidant activity, with an IC_50_ (the concentration of NPs that scavenge 50% of the initial DPPH radicals) of 62 ± 0.8 (µg/mL) for the leaves and 35 ± 0.6 (µg/mL) for the stems. NPs and precursor concentrations were modeled to assess their impact on bacteria using a 2D polynomial equation. Response surface plots identified optimal concentration conditions for antibacterial effectiveness against each species, promising in combating antibiotic resistance.

## 1. Introduction

Bacterial antimicrobial resistance (AMR), a phenomenon where bacteria evolve to reduce the effectiveness of drugs designed to cure infections, has become a significant global health challenge in the 21st century. The review on AMR, initiated by the UK Government, suggests that by 2050, AMR could be responsible for the deaths of 10 million individuals annually [1]. Therefore, the World Health Organization (WHO), along with various other organizations and researchers, concurs that the proliferation of AMR is a critical concern, demanding a unified, worldwide strategy for mitigation [1]. 

Muray et al. [1] estimated that in 2019, about 4.95 million deaths were linked to bacterial antibiotic resistance, of which 1.27 million were directly caused by bacterial AMR. Furthermore, these infections are responsible for one-quarter of deaths worldwide annually, primarily in tropical regions, such as Africa, where these diseases cause half of the deaths [2,3]. 

The rise of multidrug-resistant (MDR) and extensively drug-resistant (XDR) bacteria remains a significant public health issue due to its association with high death rates, illness, and the elevated cost of treatment [3,4,5]. These resistant strains compromise the effectiveness of existing treatments, making it imperative to explore and create new solutions to combat infections and safeguard public health. Developing these novel agents will involve innovative research and collaboration across various scientific disciplines to stay ahead of evolving bacterial threats.

As resistance to the main antimicrobial drugs grows and infections from multidrug-resistant organisms become more common, scientists are exploring new treatment alternatives. Nanotechnology has shown promising results in tackling various contemporary challenges, on its own or combined with nanobiotechnology [6].

Nanotechnology, which involves manipulating materials at the nanoscale, has advanced rapidly in various fields, providing innovative features in electronics, telecommunications, and medicine, among others. The indispensable role of nanomaterials, with their diverse properties, is evident in addressing contemporary challenges. In the realm of sustainability, green chemistry strives to prevent contamination by designing safer chemicals, and nanotechnology is actively involved in green crafting nanomaterials. These align with environmental sustainability goals through processes that are non-hazardous, energy-efficient, sustainable in synthesis, and ensure safe disposal methods [6,7]. Specifically, using extracts from medicinal plants as agents to reduce and stabilize the synthesis of ZnO nanostructures offers several benefits over traditional physical and chemical processes that can be highly toxic. The advantages of nanostructured ZnO particles compared to other metal oxide nanoparticles (NPs), such as CuO, TiO_2_, and SnO_2_, include their lower cost and lower toxicity to the human body, which enhances their biocompatibility. Additionally, ZnO NPs are known for their ability to effectively block UV rays. They also exhibit high catalytic activity and a large surface area, which are beneficial in various applications, including environmental cleanup and antimicrobial activities. ZnO NPs have shown potential in medical applications, such as antibacterial activities, anticancer treatments, agriculture enhancements, biological sensing, molecular diagnostics and theranostics, and nanomedicine discovery [8], as well as wastewater treatment. These properties make ZnO NPs a versatile and valuable material in both industrial and medical fields [9,10].

NPs provide a practical solution for addressing many bacterial infections, especially those involving MDR organisms. Whether used individually or in synergy with antibiotics, NPs can significantly enhance therapeutic outcomes, offering remarkable synergistic benefits. This innovative approach is particularly valuable in fighting infections resistant to conventional treatments [11,12]. The antimicrobial or biomedical properties of NPs largely depend on how they are synthesized and the conditions under which they are formulated. Factors such as the reducing agent used, the temperature during synthesis, the concentration of materials, and the type of solvent all play crucial roles in determining the effectiveness and characteristics of the resulting NPs [3,13,14,15].

Recently, a variety of plants have been effectively used for the quick and efficient biosynthesis of ZnO NPs from outside the cell. *Nepeta* is one of the largest genera in the *Lamiaceae* family, often referred to as Labiatae. The name “*Nepeta*” traces back to “*Nepi*”, an ancient city in Italy [16,17]. A single species within the Nepeta genus can have various local names across different regions of the same or distinct countries, potentially due to dialectical differences. Additionally, this species might be used in diverse manners to address either the same or various ailments, reflecting the rich tapestry of traditional knowledge and practices surrounding medicinal plants [17]. These plant species possess antibacterial [18,19,20,21], antifungal [22], antioxidant [23,24,25], anti-nociceptive, and anti-inflammatory [26,27,28] activity, as well as insecticidal, anti-leishmania, anti-malarial, and anti-melanogenesis activity [17]. The phytochemical composition of *Nepeta nepetella* subps. *amethystine* has been characterized by the occurrence of different phenolic compounds, such as caffeic acid [29], coniferin p-coumaric acid, ferulic acid, 4-hydroxybenzoic acid, vanillic acid, nepetoidin, nepetoidin B, rosmarinic acid, and iridoid monoterpenes and triterpenes [15].

This study distinguishes itself through the creation of predictive models designed to map the specific behaviors of bacterial species and different plant parts in reaction to changes in NP biosynthesis conditions. These conditions include the concentrations of NPs and precursors, aiming to refine the synthesis process to achieve optimal antibacterial effectiveness.

## 2. Materials and Methods

### 2.1. Material

**Figure 1:**
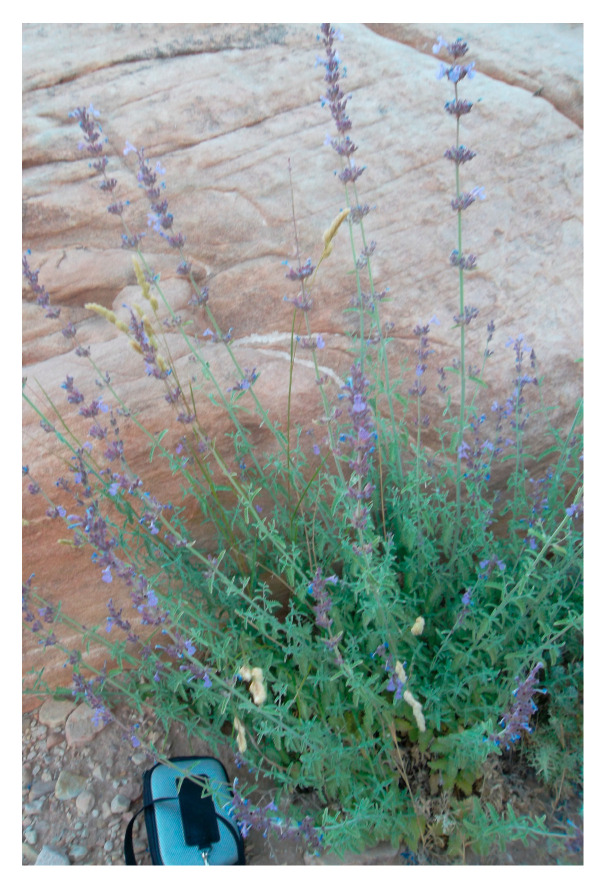
Original photo of *Nepeta nepetella* subps. *amethystine* taken at Djebel Aissa at an altitude of 1600 m (photo taken by Dr. Gordo Belkacem).

The reagents used included zinc sulfate heptahydrate (ZnSO_4_·7H_2_O, 99%) and sodium hydroxide (NaOH), provided by BIOCHEM Chemopharma, Cosne-Cours-sur-Loire, France, along with methanol (CH_3_OH, 99.9%), provided by Sigma-Aldrich, Darmstadt, Germany. Mueller–Hinton agar was purchased from Condalab (Laboratorios Conda S.A., Madrid, Spain). The strains *Staphylococcus aureus* ATCC 25923, *Listeria monocytogenes* ATCC 19115, and *Bacillus cereus* ATCC 11768, as well as Gram-negative bacteria *Escherichia coli* ATCC 25922, *Salmonella enteritidis* ATCC 13076, and *Klebsiella pneumoniae* ATCC 70603 were provided by the Laboratory for Sustainable Management of Natural Resources in Arid and Semi-Arid Areas, University Center of Salhi Ahmed (Naâma, Algeria), obtained from the Pasteur Institute (Algiers, Algeria). The plant material was collected from the Djebel Aissa National Park, Ain Sefra Commune, Naâma Province, Algeria (Figure 1). 

### 2.2. Preparation of Extracts

The leaves and stems were washed, air-dried, and finely ground into powder. Aqueous extracts of the leaves and the stems were prepared by adding 5 g of powder to 100 mL of distilled water. The mixture was then infused at 60 °C for 1 h. The resulting mixture was subsequently filtered through Whatman No. 1 filter paper. The extract was then stored at 4 °C [30].

#### Total Polyphenol Content (TPC)

The quantification of total polyphenols was conducted using the Folin–Ciocalteu method, as described by Jiménez-Rosado et al. [31]. Initially, 50 μL of the aqueous extract from the leaves and the stems of *Nepeta nepetella* subps. *amethystine* was added to 2 mL of distilled water and 250 μL of 1N Folin–Ciocalteu reagent for 8 min. Subsequently, 750 μL of 20% Na_2_CO_3_ and 950 μL of distilled water were added. After allowing the mixture to mature for 30 min in the dark, the absorbance was measured at 765 nm using a spectrophotometer. Gallic acid served as the reference standard, and the results were reported in milligrams of gallic acid equivalents (GAE) per gram of extract. 

### 2.3. Green Synthesis of Zinc Oxide Nanoparticles

A solution consisting of 40 mL of zinc sulfate with concentrations of 0.5 M, 1 M, and 2 M was prepared and thoroughly mixed. To this solution, 10 mL of the aqueous extract from the leaves and stems of *Nepeta nepetella* subps. *amethystine* was added drop-by-drop, as described by Pachaiappan et al. [30]. The entire mixture underwent vigorous stirring using a magnetic stirrer, and then the solution’s pH was adjusted to 12 with the addition of a 2 M sodium hydroxide solution [32]. The reaction was allowed to proceed for 1 h under continuous magnetic stirring. Subsequently, the solution underwent centrifugation and was washed six times for 10 min at 10,000 rpm. The resulting precipitate was dried at 70 °C overnight. Finally, the dried precipitate was subjected to calcination in a muffle furnace at 500 °C for 2 h. The resulting powder was finely ground using a mortar and pestle.

### 2.4. Characterization of Zinc Oxide Nanoparticles

The confirmation of crystalline phases in the sample was achieved by obtaining an XRD pattern using a Bruker D8 Advance A25 diffractometer featuring a Cu anode (manufactured by Bruker Corporation and sourced from Madrid, Spain), utilized not only for surface morphology but also for identifying elemental composition and confirming the dimensions and form of the green-synthesized ZnO NPs.

To evaluate the zeta potential of the prepared ZnO NPs, a 4 mg/mL ZnO NPs solution was prepared in distilled water. Then, 900 µL of the dispersion was injected into DTS1070 cells, and zeta potential measurements were conducted using the Malvern Zetasizer Nano (ZSP, Malvern, UK) at a temperature of 25 °C. The acquired data were analyzed using Zetasizer Software 8.02.

The confirmation and characterization of synthesized ZnO NPs derived from the aqueous extract of *Nepeta nepetella* subps. *amethystine* were conducted through various analytical techniques. A Fourier transform infrared spectroscopy (FTIR) SHIMADZU FTIR-8400 was utilized over the range of 4000–400 cm^−1^ to identify functional groups and phytoconstituents contributing to the reduction and stabilization of the ZnO NPs. For the preparation of the sample for FTIR analysis, 0.2 mg of potassium bromide (KBr) was mixed and ground with 0.002 mg of NPs. The mixture was then compressed into a pellet and inserted into the FTIR instrument. The measurements were obtained between 4000 and 400 cm^−1^ with a resolution of 4 cm^−1^ and an acquisition of 40 scans.

To investigate the morphology and structure of NPs, surface morphology was studied using a Zeiss EVO scanning electron microscope (Watertown, MA, USA) operating at 10 kV. The ZnO NPs were deposited onto an SEM stub using a conductive adhesive before being scanned.

The size, shape, and distribution were determined by a Talos S200 (FEI, Hillsboro, OR, USA) transmission electron microscope at 200 kV in a bright field. The ZnO NPs were initially dispersed in an ethanol dispersant solvent. Then, a small droplet of the dispersion (approximately 2 µL) was deposited onto a carbon-coated copper grid (Cu grid) and allowed to dry completely. The morphology was further analyzed using ImageJ software (v1.53e).

### 2.5. Antioxidant Activity

The antioxidant activity of green-synthesized ZnO NPs was determined using 2,2-diphenyl-1-picrylhydrazyl (DPPH) and the total antioxidant activity of phosphomolybdate (TAC) following the protocol described by Abdullah et al. [33].

For the DPPH assay, 2 mL of a 5.9 mM DPPH solution was added to 2 mL of green-synthesized ZnO NPs in water at different concentrations (12.5, 50, 125, and 250 µg/mL). These concentrations were prepared via the dilution method. Ascorbic acid was used as a standard. The control DPPH was measured without a sample. The mixture was shaken vigorously and allowed to stand at room temperature for 30 min. To measure the absorbance, a spectrophotometer was used at 517 nm. The IC_50_ value was calculated using a Log dose inhibition curve using GraphPad Prism 9. The percentage of the DPPH scavenging effect was calculated using the following equation:

DPPH scavenging effect (%) = A_0_ − A_1_/A_0_ × 100 (1)

where: 

A_0_: is the absorbance of the control reaction.

A_1_: is the absorbance in the presence of the test.

For the total analysis of phosphomolybdate (TAC) assay, 2 mL of ZnO NPs dispersed in distilled water was combined with 2 mL of a reagent mixture containing 0.6 M H_2_SO_4_, 28 mM Na_2_HPO_4_, and 4 mM (NH_4_)_3_PMo_12_O_40_. This mixture was then incubated in a water bath at 95 °C for 90 min. The absorbance was measured at 695 nm using a spectrophotometer (Specord 50 plus, Analaytik Jena AG, Jena, Germany). The TAC was determined in micrograms of ascorbic acid equivalent per milligram of ZnO-NPs (µg AAE/mg ZnO-NPs).

### 2.6. Antibacterial Activity of Zinc Oxide Nanoparticles

The antimicrobial efficacy of green-synthesized ZnO NPs was assessed against six distinct pathogenic microorganisms, including Gram-positive bacteria *Staphylococcus aureus* ATCC 25923, *Listeria monocytogenes* ATCC 19115, and *Bacillus cereus* ATCC 11768, and Gram-negative bacteria *Escherichia coli* ATCC 25922, *Salmonella enteritidis* ATCC 13076, and *Klebsiella pneumoniae* ATCC 70603. The Kirby–Bauer method was employed to determine antimicrobial activity using various concentrations of ZnO NPs. The bacterial strains were cultured at 37 °C for 18 h to prepare the working cultures. These cultures were then diluted in fresh medium to an optical density (OD 600), achieving a concentration of 10^8^ CFU/mL. A 100 µL sample from each culture was spread evenly on Mueller–Hinton Agar plates using a sterile swab. Antimicrobial susceptibility testing discs soaked with 10 µL of ZnO NP suspensions at concentrations of 10 µg/mL, 50 µg/mL, and 100 µg/mL were dried for 10 min before being placed on the surface of Mueller–Hinton agar. The plates were incubated at 37 °C for 24 h, after which the diameters of the inhibition zones were measured. Gentamycin served as the positive control in the experiments [34]. Sterile distilled water was used as the negative control.

### 2.7. Statistical Analysis

The experiments were replicated three independent times, and the data are presented as mean ± SEM (standard error of the mean). Statistical analysis of the data was carried out using ANOVA, followed by Tukey’s HSD post hoc test (equal variances). These tests were performed using R version 4.3.2. Differences were considered statistically significant when the *p* < 0.005 while also evaluating variance heterogeneity. The results were used to create a polynomial model for the optimization of the synthesis protocol. This design was appropriate for studying the quadratic response surface methodology (RMS) and for use in the construction of a second-order polynomial model using Origin Pro 2019-b.

## 3. Results and Discussion

### 3.1. Total Polyphenols Content

The calibration curve generated from the analysis of the standard (gallic acid) was linear, with y = 0.0021x + 0.512, R^2^ = 0.98, where x stands for the gallic acid concentration and y for the absorbance. The polyphenol content in both the stems and the leaves was approximately the same, with the stems showing a slightly higher content of 50.13 ± 0.7 (mg GAE/g extract), closely followed by the leaf extracts, which contained 49.64 ± 0.9 (mg GAE/g extract).

### 3.2. Characterization of the Zinc Oxide Nanoparticles

#### 3.2.1. XRD

**Figure 2:**
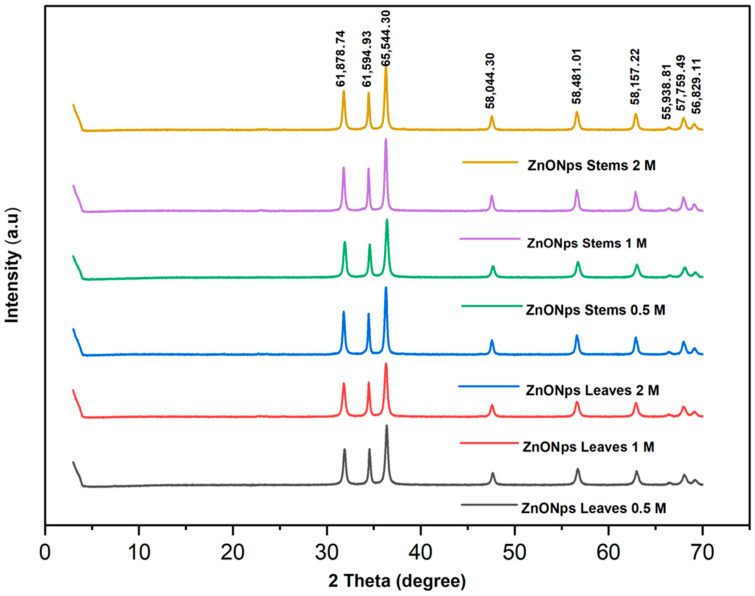
XRD spectrum of the synthesized ZnO NPs using the extract of different parts of *Nepeta nepetella* subps. *amethystine* and different precursor concentrations.

The X-ray diffractogram of the synthesized ZnO NPs is shown in Figure 2.

The crystal structures of the synthesized NPs were analyzed using XRD, revealing intense diffraction peaks at 31.87, 34.55, 36.41, 47.64, 56.70, 62.96, 66.38, 67.90, and 69.24 degrees of 2θ. These peaks could be attributed to the crystallographic reflection planes (100), (002), (101), (102), (2-10), (103), (200), (2-12), and (201), respectively, according to the Joint Committee on Powder Diffraction Studies Standards (JCPDS card number 00-230-0450). In the XRD patterns, the presence of planes (100), (002), (101), (110), and (112) affirmed the formation of a pure wurtzite structure in the ZnO NPs. The narrow and sharp peaks of the XRD patterns affirmed the fine crystalline structure of the biosynthesized ZnO NPs [35]. The average crystallite size was calculated using the Debye–Scherrer formula [36]:

D = kʎ/βcosϴ(2)

where D is the diffracting domain size, k is the correction factor (0.94), ʎ is the wavelength used (0.154178 nm), Β is the FWHM (full width at half maximum), and ϴ is the position of the main peak. Table 1 summarizes the size of the NPs using XRD, SEM, TEM, crystallinity, as well as the IC_50_.

The obtained results revealed that the difference in the grain size of NPs (D) increased with the rise in precursor concentration, likely due to a propensity for agglomeration and aggregation. Similar findings were reported in the study conducted by Abdullah et al. [33], where the size of ZnO NPs tended to increase with higher precursor and reducing agent concentrations, the latter being the plant extract. These results were further supported by Mohammadi et al. [37], who explained that this size increase occurred through the formation of anisotropic particles when the precursor concentration was elevated. Furthermore, this growth in size was attributed to the competition between zinc ions and functional elements present in the *Nepeta nepetella* plant extract, which also increased the reduction rate.

On the other hand, the crystallinity degree (%) of the ZnO NPs was calculated using Equation (3) [36]:

Crystallinity (%) = (Area of the crystalline peaks/(Crystalline peaks arear + amorphous area)) × 100(3)

The crystallinity rate significantly increased with the precursor concentration, reaching 97.19% for *Nepeta nepetella* subps. *amethystine* stems at a concentration of 2 M zinc sulfate, whereas it was 89.84% for the same sample at a concentration of 0.5 M. This is explained by the fact that at higher precursor concentrations, ZnO nanocrystals formed more extensively compared to lower concentrations, as indicated in the research studies by Pholnak et al. and Abdullah et al. [33,38].

Contrary to the results obtained by Pholnak et al. [38], the change in concentration did not lead to the formation of new compounds. In the context of this study, the optimal concentration for green synthesis of NPs, enabling the formation of reduced-size particles, was 0.5 M. At this concentration, the sizes of the NPs formed were 16.9 ± 0.3 nm for the leaves and 16.3 ± 0.1 nm for the stems.

The sizes of the NPs increased up to 17.4 ± 0.4 nm for the leaves and 20.3 ± 0.1 nm for the stems when the precursor concentration was increased to 1 M. This increase continued up to 20.3 ± 0.9 nm for the leaves and 21.6 ± 0.4 nm for the stems when the zinc sulfate concentration reached 2 M. The production process of ZnO NPs depends on various growth parameters, including the concentration of plant extract or biomass, salt concentration, growth or reaction time, temperature, and pH of the solution. Hence, it is crucial to calibrate these growth elements to achieve the desired size and shape of NPs for optimal control and application [39].

#### 3.2.2. Zeta Potential

The examination of zeta potential provides insights into the stability of NPs. Zeta potential serves as a critical parameter, indicating the level of electrostatic repulsion among charged groups situated on the surface of particles [40]. This parameter is pivotal in assessing the stability of colloidal dispersions. When particles within a suspension exhibit either highly negative or positive zeta potentials, they tend to repel each other, preventing aggregation. Conversely, aggregation tends to occur at low zeta potentials due to reduced repulsion forces among particles. Generally, NPs with zeta potentials ranging from >+30 to <−30 are considered highly stable [41,42]. 

**Figure 3:**
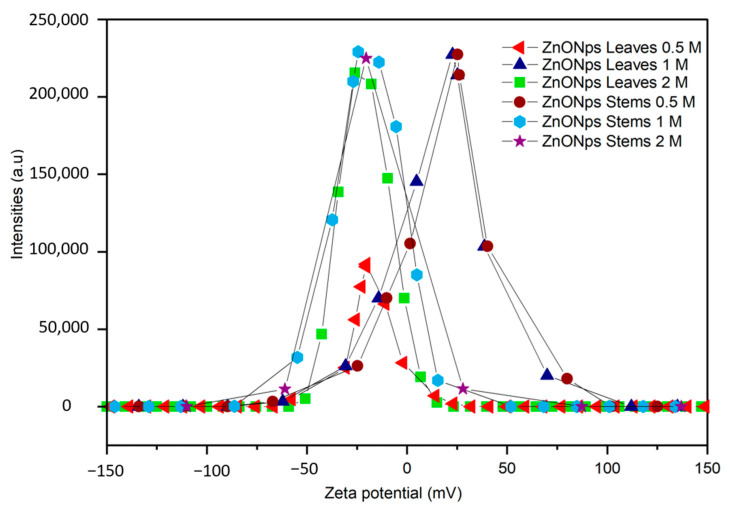
Zeta potential values of the ZnO NPs using the extracts of different parts of *Nepeta nepetella* subps. *amethystine* and different precursor concentrations (0.5 M, 1 M, and 2 M).

The zeta potential values presented in Table 2 and Figure 3 revealed significant variations based on precursor concentration and plant part. Notably, NPs derived from leaves exhibited distinct zeta potential values at different concentrations, with a marked difference observed at the 1 M concentration compared to the 0.5 M and 2 M concentrations. While stem-derived NPs showed comparable zeta potential values to leaves at the 1 M concentration, differences emerged at other concentrations. Higher zeta potential magnitudes, particularly at the 1 M concentration, suggested enhanced stability due to greater electrostatic repulsion between particles. The zeta potential values (Table 2) demonstrated both positive and negative values, indicating the surface charge of the NPs in the solution. Positive zeta potential values, such as +25.2 ± 1.5 and +25.2 ± 0 mV, suggested a predominance of positively charged groups or species on the nanoparticles’ surface. This positive charge facilitated electrostatic repulsion between particles, enhancing the dispersion stability. Conversely, negative zeta potential values, for instance, −20.4 ± 0.1 and −20.5 ± 1.8 mV, indicated the presence of negatively charged functional groups or species on the nanoparticles’ surface. These negative charges similarly promoted repulsion between particles, contributing to colloidal stability.

The observed variability underscores the importance of considering factors such as surface chemistry and size distribution in NPs’ synthesis and application. All NPs had zeta potential values between +30 mV and −30 mV, indicating an incipient stability [40,41]. According to Mudalige et al. [43], the zeta potential of NPs is influenced by various factors, including surface chemistry, particle concentration, size, pH of the medium, temperature, solvent, and ionic strength. Concluding, it is important to note that these assessments were conducted in water. Further analysis under different pH levels and other solvents may be necessary to comprehensively understand the behavior and stability of these NPs in diverse environments and facilitate their effective utilization in various applications.

#### 3.2.3. Fourier Transform Infrared Spectroscopy (FTIR)

**Figure 4:**
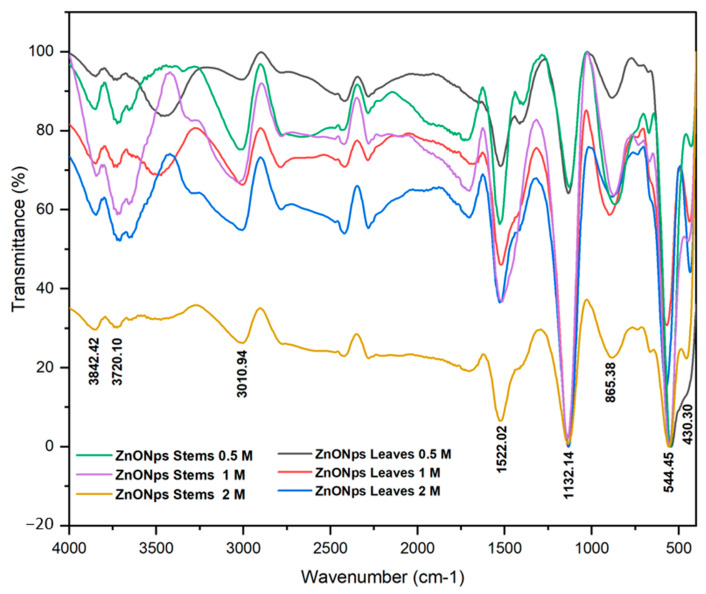
FTIR spectrum of the synthesized ZnO NPs using the extract of different parts of *Nepeta nepetella* subps. *amethystine* and different precursor concentrations.

The FTIR analysis was performed to determine the nanoparticles’ nature and purity [44] and to detect the different functional groups contributing to the synthesized NPs’ reduction, capping, and stabilization [35]. The FTIR spectrum of the synthesized ZnO NPs from *Nepeta nepetella* subps. *amethystine* is depicted in Figure 4.

Table 3 indicates the different peaks and functional groups of the ZnO NPs. The peaks at 3714–3734 cm^−1^ represented the carboxylic functional group (COOH). The absorption peaks in the range of 3500–3000 cm^−1^ were attributed to the stretching vibration of the O-H groups. This observation is consistent with the presence of alcohols, phenols, or acids in hydrogen-bonded forms [45]. The C-H group’s stretching vibrations were about 2992–3009 cm^−1^, and this bond is commonly present in organic compounds, such as terpenoids [46]. The peaks at 1517–1528 cm^−1^ were indicative of the C-H bending vibrations in aromatic rings, characteristic of the presence of aromatic compounds. The peak at 1402 cm^−1^, typical for amine or amide, represented the presence of the N-H bending of the organic compound, indicating the presence of a nitrogen-containing compound [47]. The bonds in the range of 1129–1140 cm^−1^ were assigned to C–O–H in phenolic compounds [33].

The peaks observed at 429–456 cm^−1^ and 544–573 cm^−1^ were due to the Zn-O vibrations of ZnO NPs. These identified peaks confirmed the presence of phytochemicals, such as terpenoids and phenolics, in the plant extract, suggesting their involvement in the reduction and stabilization of ZnO NPs [48]. These findings align with and are supported by previous research results [37,49].

The peaks at 865–897 cm^−1^ indicated the existence of a functional group associated with the C=C bending of alkene [50]. These FTIR findings not only confirmed the chemical composition and functional groups present in the sample but also suggested the presence of specific phytochemicals, such as terpenoids and phenolics, in the plant extract. These compounds are known for their ability to reduce metal ions to NPs and stabilize them, which, in this context, related to the formation and stabilization of ZnO NPs.

#### 3.2.4. Scanning Electron Microscopy (SEM)

**Figure 5:**
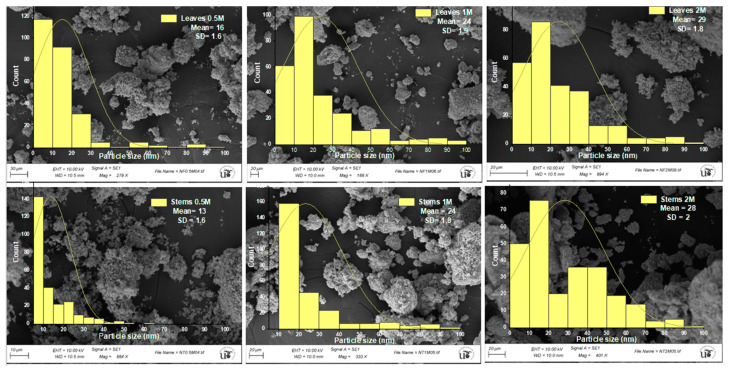
Scanning electron microscopy (SEM) of the synthesized ZnO NPs using the extracts of leaves and stems of *Nepeta nepetella* subps. *amethystine* and different precursor concentrations (0.5 M, 1 M, and 2 M).

Figure 5 displays SEM photographs of the synthesized ZnO NPs, showcasing various morphologies and histograms depicting the size distributions of the NPs.

The SEM images disclosed the distinctive spherical shape, coexisting with a nanoflake shape, of ZnO NPs, and similar morphologies were reported by Gurgur et al. [51]. The particle size varied with the concentration of zinc sulfate. For the lowest concentration of 0.5 M, sizes ranged from 13.7 ± 1.6 nm to 16.3 ± 1.6 nm, for a concentration of 1 M, sizes were 24.2 ± 1.9 nm to 23.5 ± 1.8 nm, and for the highest concentration of 2 M, sizes spanned from 28.4 ± 2 nm to 29.3 ± 1.8 nm, for leaves and stems, respectively. We could distinguish NPs with a very clear spherical shape, unlike at higher concentrations (1 M and 2 M of zinc sulfate), where more clustered and aggregated NPs could be observed, making it difficult to discern the nanoparticle shapes. The phenomenon of agglomeration observed in NPs systems can often be attributed to the calcination temperature (500 °C), as reported in the study conducted by Chan et al. [52], where different temperatures were utilized in the green synthesis of Cu NPs, revealing a propensity for NP agglomeration and particle size with increasing calcination temperature. Furthermore, at 600 °C, aggregation led to the disappearance of the grain boundary area, attributed to disturbances caused by grain growth, resulting in a decrease in crystal surface energy during aggregation under calcination. It was observed in the same study that the NPs exhibited a spherical shape at a calcination temperature of 500 °C.

The interaction of NPs with each other may lead to aggregation. In other cases, aggregation can be due to the phytochemical compounds of the plant, which may be the case here, as the FTIR results indicated the presence of phytochemical compounds on the surface of the NPs. Also, the presence of H-bonding in the bioactive molecules would lead to aggregation [33,53,54]. The phytochemicals present in these plant extracts possessed both reducing and antioxidant properties. Manipulating the shape and size below and above the micellar concentration facilitated the growth and stability of NPs. Consequently, this material can serve as a template/surfactant for the nucleation and controlled growth of NPs, allowing for the regulation of morphology, stabilization of size, and adjustment of various properties. This approach enables the preparation of NPs tailored for specific applications [55]. Overall, these bioactive compounds have so far acted as reducing agents, capping agents, and stabilizing agents for the metal oxide NPs [36,56,57,58].

According to classical nucleation and growth theory, the concentration of precursors plays a pivotal and immediate role in influencing both the nucleation and growth rates. This is because the reaction can occur under either thermodynamic or kinetic growth control regimes. Nuclei growth under dynamic control generates nano- and micro-particles with anisotropic shapes and varying dimensions. These formations result from differences in the growth velocities of different crystal facets. The relative growth rates of these crystal faces ultimately dictate the final shape and aspect ratio of the ZnO structures [45]. According to Abdullah et al. [32], in addition to reaction kinetics and the nucleation rate, the solubility of the precursor salt is crucial for influencing the formation, size, surface charge, and morphology of NPs by promoting rapid nucleation and crystal growth.

#### 3.2.5. Transmission Electron Microscopy (TEM)

**Figure 6:**
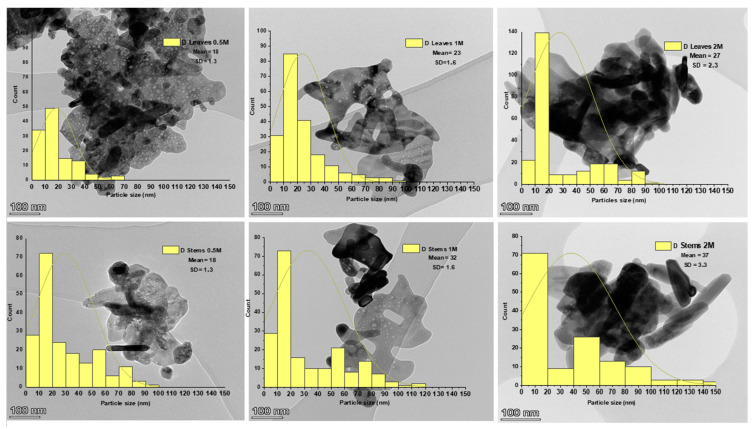
Transmission electron microscopy (TEM) of the synthesized ZnO NPs using the extracts of leaves and stems of *Nepeta nepetella* subps. *amethystine* and different precursor concentrations (0.5 M, 1 M, and 2 M).

Figure 6 displays TEM photographs of the synthesized ZnO NPs, showcasing various morphologies and histograms depicting the size distributions of the NPs.

The TEM images revealed spherical aggregate ZnO NPs, with an average size of 18.1 ± 1.3 nm and 18.5 ± 1.3 nm for both stems and leaves for the lowest concentration of the precursor, displaying an absence of nanorods. Conversely, for the highest concentrations of the precursor (1 M and 2 M), the NPs appeared spherical and more agglomerated, incorporating nanorods (Figure 6), with an average size of 23.3 ± 1.6 nm and 32.1 ± 1.6 nm at 1 M and 27.7 ± 2.3 nm and 37.1 ± 3.3 nm at 2 M for the leaves and the stems, respectively. NPs of a similar size scale have been obtained in various green synthesis studies of ZnO NPs [33,35,47]. According to Barzinjy et al. [39], agglomeration is frequently observed in green synthesis NPs. This behavior is linked to the increased surface area of biosynthesized NPs and their strong affinity, resulting in aggregation or agglomeration. It can be affirmed that ecological factors play a substantial role in influencing the stability and agglomeration of NPs. Ecological factors significantly impact the stability and agglomeration of NPs. Ecological conditions, such as temperature, pH, and the presence of ions or organic matter, can substantially influence NPs’ behavior. Temperature changes can increase NPs’ kinetic energy, possibly leading to enhanced agglomeration. Similarly, pH adjustments can affect their surface charge, impacting stability and aggregation. Moreover, salts or organic compounds can prompt aggregation by modifying electrostatic and steric interactions among particles. Consequently, during the process of NP formation, particles adhere to each other and spontaneously form asymmetrical clusters [39].

NPs with the smallest sizes and the most uniform distribution were obtained during synthesis with the lowest concentration of zinc sulfate, which was 0.5 M. This could indicate that the ratio between the number of polyphenolic compounds present in the plant and the precursor concentration was optimal in this case [59,60]. The growth mechanism of ZnO NPs using zinc sulfate (ZnSO_4_·H_2_O) and *Nepeta nepetella* subps. *amethystine* extract involved a chemical reaction that can be described by the following equation [32]:

nZnSO_4_^−2^ + 2nR-OH → nZnO + nH_2_O + 2nR-SO_4_^−2^ (4)

where R-OH represents the phenolic compounds of the plant extract.

Zayed et al. [60] highlighted that when the volume of the extract is not in proportion to the precursor concentration, the irregularity in size, shape, and size distribution of NPs becomes apparent. This phenomenon was observed in our study as we increased the precursor concentration beyond 0.5 M. Furthermore, in cases of excess extract volume, there was a significant tendency for NPs to take on round shapes. These results were consistent with the findings of Abdullah et al. [33], who elucidated that when the precursor quantity is lower than that of the extract, NPs tend to aggregate strongly, forming cubic structures due to the competition between the functional groups of the plant and zinc ions. Conversely, when the extracted quantity is substantially higher than that of the precursor, NPs exhibit improved dispersion and assume more diverse shapes [60]. Park et al. [61] affirmed that the concentration of the precursor affects the size of the particles. When the precursor concentration is low, the supply rate of the zero-valence metal ions required for nucleation is slow, and the critical size needed for nucleation increases. In contrast, when the precursor concentration is sufficient, the critical size for nucleation decreases because the supply rate of zero-valence metal ions required for nucleation is high. Accordingly, when the precursor concentration is significantly low, the particles become large, and when the precursor concentration is sufficient, small and uniform particles can be obtained. 

### 3.3. Antioxidant Activity

**Figure 7:**
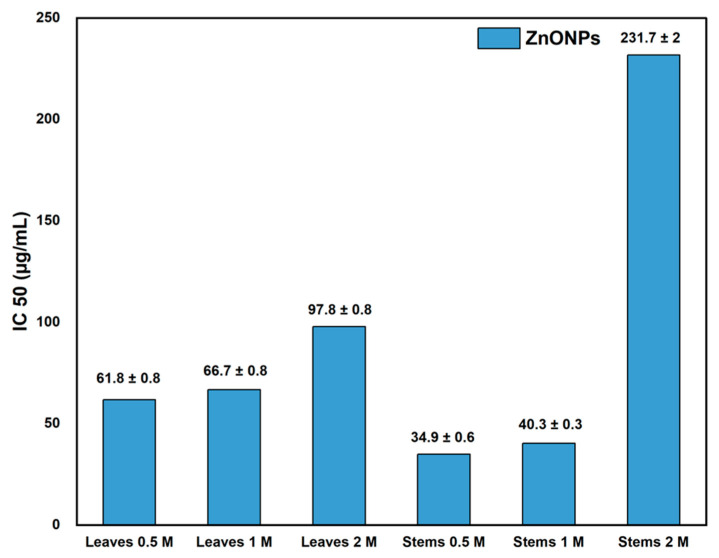
DPPH IC_50_ values (µg/mL) for the ZnO NPs from the different parts of the plant (leaves and stems) and the different precursor concentrations (0.5 M, 1 M, and 2 M).

The synthesized ZnO NPs exhibited antioxidant activity, quantified by the IC_50_ inhibition percentage, as illustrated in Table 1 and Figure 7. Figure 7 displays the IC_50_ values of ZnO NPs derived from the leaves and stems of *Nepeta nepetella* subps. *amethystine* produced using different precursor concentrations (0.5 M, 1 M, and 2 M). The IC_50_ value represents the sample amount required to scavenge 50% of free radicals.

The best antioxidant activity was determined with an IC_50_ of 34.9 ± 0.6 (µg/mL) for ZnO NPs from stems synthesized with the lowest precursor concentration of 0.5 M. In contrast, the lowest antioxidant activity was observed with an IC_50_ of 231.7 ± 2 µg/mL for ZnO NPs from *Nepeta nepetella* subps. *amethystine* stems at a precursor concentration of 2 M. NPs synthesized at the lowest precursor concentration of 0.5 M exhibited the highest antioxidant activity, likely due to their small size and the presence of capping agents. In contrast, larger NPs showed lower antioxidant activity [9,33].

The IC_50_ values for ZnO NPs were found to be higher than that of ascorbic acid (1.53 µg/mL), indicating that ascorbic acid was more effective in scavenging DPPH free radicals compared to ZnO NPs. These findings were significantly higher than the IC_50_ values for the aqueous extract of *Nepeta nepetella* subps. *amethystine*, which was 850 µg/mL for stems and 2000 µg/mL for leaves. Many studies showed a significant positive correlation between the phenolic compounds contained in herbal extracts [62] or other plant components, such as lignin, and their antioxidant activity [63,64]. However, in this study, the levels of polyphenolic compounds in the leaves and stems did not exhibit significant differences. The variation in antioxidant potential between the leaves and stems could be attributed to the presence of other plant compounds, such as flavonoids or terpenoids. These compounds may also act as reducing agents in the NP biosynthesis process, along with proteins, ketones, sugars, and carbohydrates [65]. Additionally, considering the intricate composition of phytochemicals, particularly considering that the assessment of antioxidant activity heavily depends on the reaction mechanism, employing various approaches for the measurement of the antioxidant activity may provide insightful results [66].

The antioxidant activity of ZnO NPs is commonly attributed to the small size of the particle grains. Another reason could be related to the phenomenon of electron density transfer from the oxygen atom to the unpaired electron located on the nitrogen atom in DPPH, resulting in a decrease in the intensity of the n→π∗ transition [9]. 

NPs with antioxidant properties have various potential applications across different fields, including in food, where NPs with antimicrobial and antioxidant properties show promise in extending the shelf life of food products. By combating enzymatic, oxidative, and microbial spoilage, these materials help maintain the freshness and quality of foods. Antioxidants within the nanocomposites mitigate the development of off-flavors and enhance color stability. Additionally, strategies such as using high-barrier packaging and creating anaerobic atmospheres reduce food oxidation, while active packaging with oxygen scavengers further aids in preservation. In summary, nanocomposites with dual antimicrobial and antioxidant capabilities offer effective means to enhance food preservation and prolong shelf life [67]. They also hold materials for gene delivery, biomedical applications, and therapy for different environmental pollutant-induced toxicity [68].

Regarding the total antioxidant capacity (TAC) assay, it relies on the conversion of Mo(VI) to Mo(V) via the action of the sample analyte, producing distinct green phosphate Mo(V) compounds [69]. The TAC of synthesized ZnO NPs using different parts of *Nepeta nepetella* subsp. *amethystina* varied with the concentration of the precursor and the plant part used, as shown in Table 1. While all samples displayed antioxidant activity against the TAC solution across all systems, significant differences were particularly noted at the 1 M concentration for stems. Specifically, at the 0.5 M concentration, both leaves and stems showed similar TAC values, approximately 19.95–19.96 µg AAE/mg ZnO NPs, indicating no significant differences. This suggests that at lower concentrations, the precursor’s effect on TAC was minimal, regardless of the plant part used. 

At the 1 M concentration, a significant increase in TAC was observed, especially for stems, which reached up to 22.70 µg AAE/mg ZnO NPs. This value surpassed that of leaves at the same concentration (20.57 µg AAE/mg ZnO NPs), highlighting a notable difference in antioxidant capacity at this intermediate concentration. However, at the 2 M concentration, the TAC decreased for both leaves and stems. Although stems still exhibited slightly higher TAC (19.28 µg AAE/mg ZnO NPs) compared to leaves (18.54 µg AAE/mg ZnO NPs), the differences were not significant, suggesting a plateau or decline in TAC with higher precursor concentrations. Again, the lack of significant differences at this concentration may be due to the aforementioned factors influencing the overall antioxidant capacity. Therefore, in summary, although the TAC differed in the concentration of the precursor and plant part used, it was basically at the 1 M concentration that the main difference was recorded in stems. Stems generally demonstrated higher TAC than leaves, with the highest antioxidant activity observed at the 1 M precursor concentration. These results underscore the intricate interplay of factors, such as reactive oxygen species (ROS) generation, NP size, crystallinity, and stability, which collectively influence the observed outcomes [32]. 

### 3.4. Antibacterial Activity

An initial analysis was conducted using a one-way ANOVA followed by a Tukey’s test to assess the significance level of the combination of the factors (NP concentration, precursor concentration, and plant part) on the inhibition zone for each bacterial species. This analysis revealed a high level of significance, indicating the impact of these factors on antibacterial effectiveness.

The obtained results (Table 4) enabled the identification of the most effective NP concentrations against each bacterial species (Table 5). 

A second analysis was performed using a three-way ANOVA to assess the interaction between each different parameter: the concentration of NPs, the part of the plant used, and the precursor concentration. This was carried out to determine if any of these factors, or an interaction among them, impacted antibacterial activity. The obtained results (Table 6) indicated a statistically significant difference between the different groups, suggesting that the variation closely influenced the inhibition zone in experimental conditions. Tukey’s test results for the “NP concentration” factor in relation to the other factors (CP and PP) showed a statistically significant difference between the varying concentrations of 10 µg/mL, 50 µg/mL, and 100 µg/mL on the inhibition zone variation. 

The analysis showed that concentrations of 10 µg/mL and 50 µg/mL offered the best antibacterial effect across both Gram-positive and Gram-negative species compared to the 100 µg/mL concentration, demonstrating the worst effect. This aligned with the mean comparisons obtained by the one-way ANOVA (Table 4). 

Based on these findings, it was observed that the most effective antibacterial activity predominantly arose from NP concentrations of 10 µg/mL and 50 µg/mL. This outcome could be interpreted by considering various factors:

(i) Particle size and size distribution. The influence of NP size and size distribution on antibacterial activity was highlighted by the observation, as measured by TEM, that NPs sourced from the 1 M precursor concentration with sizes ranging between 23.3 ± 1.6 nm and 32.1 ± 1.6 nm showed the most significant effect. This contrasted with ZnO NPs derived from the 2 M precursor, which exhibited sizes between 27.7 ± 2.3 nm and 37.1 ± 3.3 nm. Wu et al. [70] achieved a size-controlled synthesis of Ag NPs, ranging from 2 nm to 32 nm, by adjusting the pH to 11, 9, and 7, respectively, using sodium borohydride as a reducer and sodium citrate as a stabilizer. Antibacterial tests against both Gram-negative *E. coli* and Gram-positive *S. aureus* showed enhanced effects with smaller NPs. Particularly, 2 nm particles exhibited the most potent antibacterial activity. Yamamoto et al. [71] also found that the size of ZnO NPs (100–800 nm) affected their antibacterial activity against *S. aureus* and *E. coli*. By measuring electrical conductivity associated with bacterial growth, they concluded that a reduction in particle size increased antibacterial activity. Raghupathi et al.’s [72] research on *E. coli* and *S. aureus*, using NPs of various sizes, aligns with the findings that smaller NPs exhibited enhanced antibacterial properties. On the other hand, the impact of NPs’ size on the electrochemical gradient, established by the movement of hydrogen ions through the cell membrane, facilitating the diffusion of metal ions, was significant. Smaller NPs exhibited enhanced electrostatic interactions [73]. The study of antibacterial activity conducted by Abdullah et al. [33] on ZnO NPs revealed that smaller-sized NPs, measuring 18.6 nm, exhibited a more effective antibacterial effect than larger-sized ones, measuring 28.5 nm. Specifically, for *Staphylococcus aureus*, the 18.6 nm NPs demonstrated an antibacterial effect of 19.4 nm, while for *Escherichia coli*, this effect was 21 nm. Conversely, the 28.5 nm NPs showed less pronounced antibacterial effects, with values of 18.2 nm for *Staphylococcus aureus* and 17.4 nm for *Escherichia coli*. These results underscore the significant impact of ZnO NPs’ size on their antibacterial activity, indicating a trend toward increased efficacy with smaller sizes.
(ii) The surface-to-volume ratio. The surface area of a nanoparticle is influenced by its shape, size, and material composition. Smaller NPs and those with various shapes, such as spheres, rods, and cubes, have a higher surface-to-volume ratio, affecting their surface interactions differently. This characteristic significantly impacts their chemical reactivity and biological interactions, including antibacterial efficacy [74,75,76]. The reasons why an increased surface area enhances toxicity include, firstly, the facilitation of adsorption and binding of compounds to surfaces and, secondly, the correlation between an increased surface area and the heightened production of reactive oxygen species (ROS) [74,77,78].
(iii) The shape of NPs. The research conducted by Woźniak et al. [79] found that the cytotoxicity of gold NPs (Au NPs) depends on their size and shape, which influences cellular membrane integrity and cell viability. Specifically, nanospheres and nanorods were more toxic compared to star-, flower-, and prism-shaped structures due to their smaller size and propensity to aggregate. During this study, it was observed that the NPs primarily exhibited a spherical shape, yet the emergence of nanorods became noticeable as the precursor concentration increased, leading to aggregation [80]. Moreover, the surface properties of NPs, such as being hydrophobic, hydrophilic, lipophilic, or lipophobic, are determined by their surface characteristics. These properties significantly influence how NPs interact with biological systems, including their solubility, stability, and ability to interact with cell membranes, impacting their biomedical applications and effectiveness [62]. In the study published by Motelica et al. [81], the shape of the NPs was influenced by the solvent used in their synthesis. The use of butanol led to the formation of the smallest NPs, with a rod shape, exhibiting the highest antibacterial activity. This shape is believed to be responsible for the effective penetration of the NPs into the cell membranes.

Other factors define the properties of NPs and, thus, their toxicity, such as chemical and electronic composition, structure, as well as topology [82]. Aggregation and concentration also influence toxicity, as they lead to an increase in size, as well as cellular age, temperature, pH, and reaction time, as indicated by the study conducted by Mahsa et al. [83], where silver NPs lost their effectiveness after 48 h of contact with *Staphylococcus aureus* [3,84,85].

The mechanism of action of ZnO NPs on bacterial cells has not been precisely described, but several mechanisms are proposed: (i) The production of ROS (reactive oxygen species) includes the superoxide anion (O_2_^−^•), hydrogen peroxide (H_2_O_2_), and hydroxide (OH^−^) [72,76,86]. Raghupathi et al. [72] explained the generation of ROS, as follows: Since ZnO with defects can be activated by UV and visible light, electron–hole pairs (e^−^h^+^) can be created. The holes split the H_2_O molecules (from the ZnO suspension) into OH− and H^+^. Dissolved oxygen molecules are transformed into superoxide radical anions (•O_2_^−^), which then react with H^+^ to generate radicals (HO_2_^−•^), which, after subsequent collision with electrons, produce hydrogen peroxide anion (HO_2_^−^). They then react with hydrogen ions to produce H_2_O_2_ molecules. The generated H_2_O_2_ can penetrate the cell membrane and kill bacteria. Meanwhile, other research reported the production of these ROS under dark conditions [87,88]. In the study conducted by Dong et al. [78] to assess the extent of ROS-related damage, various sizes and concentrations of silver NPs were introduced. The findings revealed that bacterial DNA damage was more pronounced at a concentration of 0.5 μg/mL compared to 0.8 μg/mL. This suggests that ROS generated by bacteria can induce DNA damage, with higher ROS concentrations resulting in more severe damage. (ii) Chemical and physical interactions between the cell membrane and Zn^+2^ ions from the NPs result in the loss of cellular integrity. These include physically blocking cell membrane transport channels, causing physical damage to membrane envelope components through abrasion, penetrating the cell envelope to interact with the cell interior, directly interacting with bacterial cell envelope components via electrostatic effects, or a combination of these physical and chemical interactions [84,89,90]. (iii) Cellular internalization [91]. The mechanisms of action also vary depending on the nature of the NPs and the species tested. In the study conducted by Gouyau et al. [92] on the antibacterial potential of silver and Au NPs, the results demonstrated low activity against *Staphylococcus aureus* and *Escherichia coli* for Au NPs, whereas strong activity was observed for silver NPs. Therefore, the authors proposed two hypotheses: either the two types of NPs do not have the same mechanism of action, or the mechanism of action of each type of nanoparticle depends on the structure and composition of the cell wall of the tested species. This was exemplified by nickel NPs, which showed a stronger antibacterial activity against Gram-positive bacteria than against Gram-negative ones [93]. Alternatively, aluminum NPs exhibited robust activity against both types [94].

Finding the perfect concentration, especially when using active substances such as NPs in biomedical or environmental applications, is crucial to maximize efficacy, minimize toxicity, and prevent resistance. Among the bacterial adaptation reactions, the hormesis process can be mentioned. This phenomenon has been noted when exposed to sub-lethal concentrations of reactive oxygen species (ROS) [95]. Additionally, the overexpression of extracellular proteins by bacteria, such as flagellin, can lead to the formation of an extracellular matrix that results in the aggregation of NPs [96,97]. Graves et al. [98] also reported that exposing microbes to non-lethal concentrations of NPs can facilitate an increase in resistance due to the development of mutations, leading to the overexpression and under-expression of many genes [3].

The adjustment of the second-degree polynomial model was carried out through ANOVA, with a high F-value and low *p*-value (*p* < 0.005), suggesting that the coefficients were highly significant, demonstrating that the developed model provided the best fit [99].

**Figure 8:**
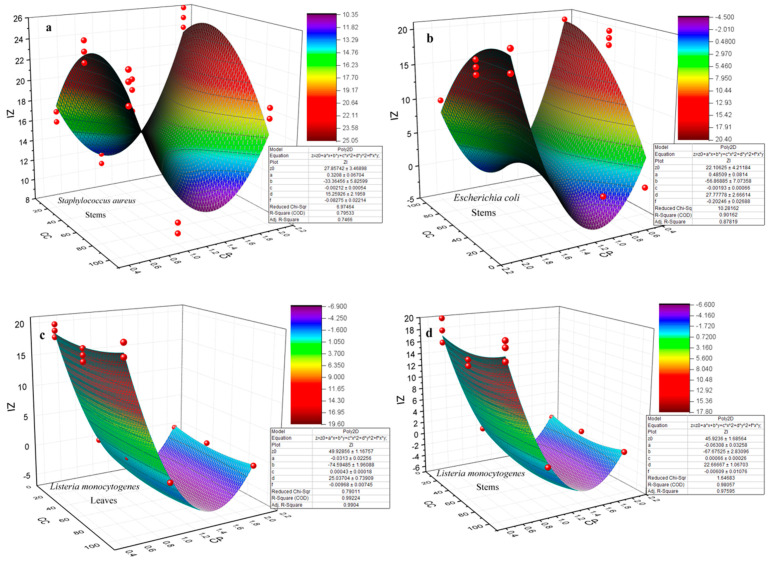

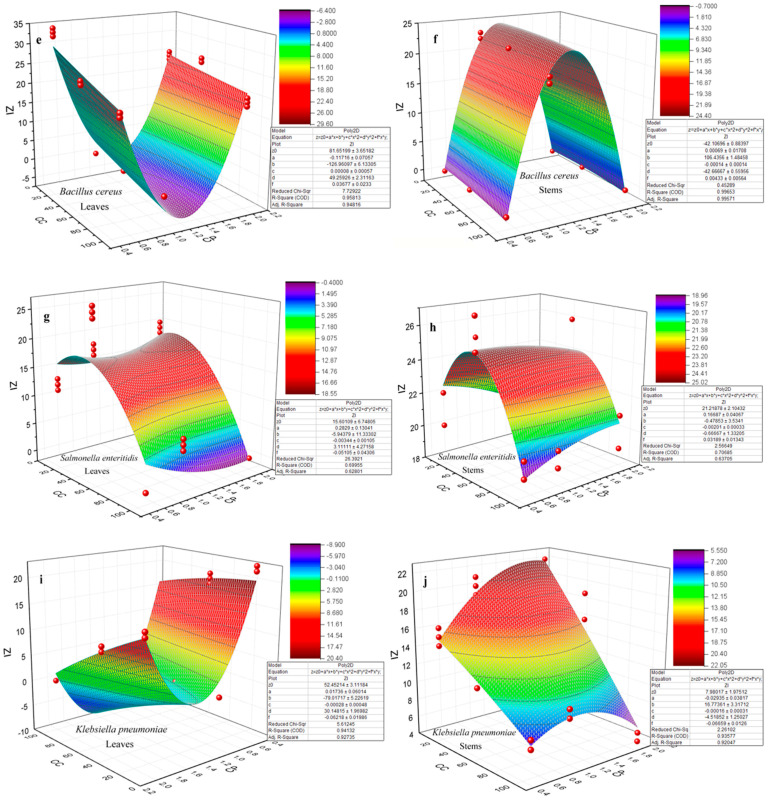
Response surface plots (3D) showing the interactive effect of variables (CC = nanoparticle concentration and CP = precursor concentration) on the inhibition (ZI = inhibition zone) of each bacterial species and for each part of the plant *Nepeta nepetella* subps. *amethystine* (**a**–**j**).

The response surface methodology (RSM) created based on the adopted polynomial model, as well as the representation expressed by the equations (Figure 8), can be considered an insightful approach. Additionally, they demonstrated effectiveness in analyzing the interactions between two factors alongside the influence of the experimental variables on the responses for a better understanding of the reaction system.

The evaluation of the model’s quality, which relied on the correlation coefficient and standard deviation, underscores the precision of the model’s predictions. The effectiveness of the model in predicting the response was reflected in a low deviation and a correlation coefficient nearing R^2^ [99].

Figure 8 displays the response surface for two variables. It can be observed that the value of the inhibition zone (IZ), depending on the concentration of NPs (CC) and the concentration of the precursor (CP), is illustrated by 3D graphical representations of the response surface for each bacterial species and each part of the plant, except for the species *Staphylococcus aureus* and *Escherichia coli* for the leaves part, where the coefficient of determination R^2^ was at 41% and 39%, respectively.

The optimized conditions for achieving the most effective antibacterial activity against each bacterial species, as determined by the models, were as follows:
Staphylococcus aureus ATCC 25923Stems = 37.5 µg/mL, 2 MEscherichia coli ATCC 25922Stems = 20.02 µg/mL, 2 MBacillus cereus ATCC 11768Leaves = 10 µg/mL, 0.5 M Stems = 21.02 µg/mL, 1.2 MSalmonella enteritidis ATCC 13076Leaves = 26.5 µg/mL, 2 M Stems = 46.7 µg/mL, 0.8 MListeria monocytogenes ATCC 19115Leaves = 100 µg/mL, 0.5 M Stems = 100 µg/mL 0.5 MKlebsiella pneumoniae ATCC 70603Leaves =11.83 µg/mL, 0.5 M Stems = 10 µg/mL, 1.7 M


## 4. Conclusions

During this study, we varied key parameters in the green synthesis process of ZnO NPs to optimize the production of the most effective NPs against bacteria. Our findings, which aligned with several other studies, indicated that the smallest NPs demonstrated superior biological activities. Our results also showed that spherical NP sizes of 18.1 ± 1.3 nm and 32.1 ± 1.6 nm exhibited enhanced antioxidant activity, with IC_50_ values of 34.9 µg/mL and 40 µg/mL, respectively. These NPs were obtained from the precursor concentrations of 0.5 M and 1 M, respectively. In contrast, NPs from higher precursor concentrations of 2 M, with sizes of 27.7 ± 2.3 nm obtained from the leaves and 37.1 ± 3.3 from the stems, displayed IC_50_ values of 97.8 µg/mL and 231.7 µg/mL, respectively. In terms of antibacterial activity, it was noted that the smallest NPs showed a more effective action against both Gram-positive bacteria *Staphylococcus aureus*, *Listeria monocytogenes*, and *Bacillus cereus*, and Gram-negative species *Escherichia coli*, *Salmonella enteritidis*, and *Klebsiella pneumonia*. Additionally, varying NP concentrations (10 µg/mL, 50 µg/mL, and 100 µg/mL) uncovered a notable antibacterial potential at the lowest concentration of 10 µg/mL for all species tested, except *S. enteritidis*, which responded better at 50 µg/mL. The influence of NPs and precursor concentrations on each plant part and against each bacterial species was modeled through a 2D polynomial equation and visually represented on a response surface plot. This method facilitated the identification of optimal conditions for NPs and precursor concentrations to achieve the maximum antibacterial effect for each bacterial species. These findings offer a promising alternative in the fight against bacterial resistance. Additionally, the application of these ZnO NPs may extend to other biological activities, such as antiparasitic, antidiabetic, and cytotoxic activities, as well as to other areas of healthcare, such as drug delivery carriers, diagnostic imaging, biosensing, and vaccine vectors. The principal limitation of this study involved controlling for additional experimental parameters, such as the exact size and shape of NPs. Further investigations could also explore other effects, including cytotoxic or antiproliferative potentials.

Future research should also aim to track bacterial growth kinetics to ascertain the NPs’ duration of action and clarify their inhibition nature, along with incorporating more factors to refine the established model.

## Figures and Tables

**Table 1:** Results were obtained for different parameters of the ZnO NPs synthesized using different parts of *Nepeta nepetella* subps. *amethystine* extract with different concentrations (0.5 M, 1 M, and 2 M): mean diameter (of XRD crystallite size, TEM, and SEM NPs’ size), crystallinity percentage, antioxidant activity (IC_50_ DPPH free radical), and total antioxidant activity (TAC; µg AAE/mg ZnO NPs).

Concentration of Precursor	Part of the Plant	D (XRD) (nm)	D (TEM) (nm)	D (SEM) (nm)	Crystallinity (%)	IC_50_ (µg/mL)	TAC (µg AAE/mg ZnO NPs)
0.5 M	Leaves	16.9 ± 0.3 ^b^	18.5 ± 1.3 ^b^	16.3 ± 1.6 ^b^	91.0 ^b^	61.8 ± 0.8 ^c^	19.95 ± 0.7 ^ab^
	Stems	16.3 ± 0.1 ^a^	18.1 ± 1.3 ^a^	13.7 ± 1.6 ^a^	89.8 ^a^	34.9 ± 0.6 ^a^	19.96 ± 0.9 ^ab^
1 M	Leaves	17.4 ± 0.3 ^c^	23.3 ± 1.6 ^c^	24.2 ± 1.9 ^d^	91.1 ^b^	66.7 ± 0.8 ^d^	20.57 ± 0.7 ^b^
	Stems	20.3 ± 0.1 ^d^	32.1 ± 1.6 ^e^	23.5 ± 1.8 ^c^	96.3 ^c^	40.3 ± 0.3 ^b^	22.70 ± 0.9 ^c^
2 M	Leaves	20.3 ± 0.9 ^d^	27.7 ± 2.3 ^d^	29.3 ± 1.8 ^f^	93.6 ^d^	97.8 ± 0.8 ^d^	18.54 ± 1.1 ^a^
	Stems	21.6 ± 0.4 ^e^	37.1 ± 3.3 ^f^	28.4 ± 2.0 ^e^	97.2 ^e^	231.7 ± 2 ^e^	19.28 ± 0.5 ^ab^

Note: Different superscript letters within a column in a time row indicate significant differences among mean observations (*p* < 0.05).

**Table 2:** Zeta potential values of the different ZnO NP samples using different precursor concentrations (0.5 M, 1 M, and 2 M).

Nanoparticles	Leaves 0.5 M	Leaves 1 M	Leaves 2 M	Stems 0.5 M	Stems 1 M	Stems 2 M
Zeta potential (mV)	−20.4 ± 0.1 ^b^	+25.2 ± 1.5 ^a^	−20.5 ± 1.8 ^b^	+25.2 ± 0 ^a^	−24.3 ± 1.2 ^c^	−20.5 ± 1.6 ^b^

Note: Different superscript letters ^(a–c)^ within a column in a time row indicate significant differences among mean observations (*p* < 0.05).

**Table 3:** Comparative table of functional groups of ZnO NPs from different parts of the plant (leaves and stems) derived from various precursor concentrations (0.5 M, 1 M, and 2 M) according to their FTIR peaks (cm^−1^).

Functional Groups	Stems 0.5 M	Leaves 0.5 M	Stems 1 M	Leaves 1 M	Stems 2 M	Leaves 2 M
COOH	3714	3734	3732	3720	3727	3724
O-H stretching	3348	/	/	3492	/	/
C-H stretching	3009	3003	3006	2992	3003	3006
C-O-H bending	1528	1525	1517	1523	1520	1528
C-O stretching	1129	1140	1137	1134	1132	1129
C-C bending	878	878	897	873	878	865
ZnO vibration	573	556	551	548	544	548
	429	456	432	440	439	428

**Table 4:** Inhibition zones (in mm) of the synthesized ZnO NPs (ZnO NPs) against *Staphylococcus aureus* (*S. aureus*), *Listeria monocytogenes* (*L. monocytogenes*), *Bacillus cereus* (*B. cereus*), *Escherichia coli* (*E. coli*), *Salmonella enteritidis* (*S. enteritidis*), and *Klebsiella pneumonia* (*K. pneumonia*). Gentamicin was used as a positive control.

Parameters			*S. aureus*	*L. monocytogenes*	*B. cereus*	*E. coli*	*S. enteritidis*	*K. pneumoniae*
Precursor C (M)	Part	ZnO NPs C (µg/mL)						
0.5	Leaves	10	0.0 ^n^	14.3 ± 0.5 ^e^	32.8 ± 1 ^j^	09.7 ± 0.5 ^ce^	12.0 ± 1 ^n^	22.6 ± 0.5 ^fk^
0.5	Leaves	50	11.0 ± 1.4 ^f^	10.0 ±1.0 ^c^	23.3 ± 0.5 ^bd^	00.00 ^a^	26.0 ± 1 ^d^	19.0 ± 1 ^bcef^
0.5	Leaves	100	17.5 ± 0.7 ^de^	13.3 ± 1.5 ^e^	20.6 ± 0.5 ^ef^	00.00 ^a^	0.0 ^f^	14.0 ± 0 ^bd^
0.5	Leaves	Gentamicin	30.0 ± 1.4 ^ag^	38.3 ± 0.5 ^f^	39.3 ± 0.5 ^g^	33.5 ± 0.7 ^f^	31.0 ± 1 ^hi^	30.0 ± 1 ^i^
0.5	Stems	10	16.5 ± 0.7 ^d^	19.0 ± 1.0 ^di^	0.0 ^a^	00.00 ^a^	21.3 ± 1.1 ^ckl^	15.0 ± 1 ^bcd^
0.5	Stems	50	24.0 ± 1.4 ^lm^	17.0 ± 1.0 ^i^	0.0 ^a^	19.0 ± 1 ^hi^	25.6 ± 1.1 ^dm^	11.0 ± 0 ^dg^
0.5	Stems	100	22.5 ± 2.1 ^jlm^	20.3 ±1.1 ^bd^	0.0 ^a^	20.0 ± 0 ^h^	19.3 ± 0.5 ^ac^	07.6 ± 0.5 ^gh^
0.5	Stems	Gentamicin	33.0 ± 2.8 ^ab^	39.7 ± 0.5 ^f^	37.6 ± 0.5 ^g^	30.0 ± 1 ^gj^	35.0 ± 1 ^j^	27.0 ± 0 ^ik^
1	Leaves	10	31.5 ± 0.7 ^ab^	0.0 ^a^	0.0 ^a^	0.0 ^a^	17.0 ± 1 ^ab^	0.0 ^a^
1	Leaves	50	28.0 ± 1.4 ^gh^	0.0 ^a^	0.0 ^a^	11.0 ± 0 ^cde^	09.0 ± 1 ^e^	0.0 ^a^
1	Leaves	100	05.5 ± 0.7 ^k^	0.0 ^a^	0.0 ^a^	12.0 ± 1 ^bd^	06.0 ± 1 ^g^	0.0 ^a^
1	Leaves	Gentamicin	34.0 ± 0.0 ^b^	29.3 ±1.1 ^g^	29.3 ± 0.5 ^h^	28.7 ± 0.5 ^g^	29.0 ± 0 ^h^	36.0 ± 1 ^j^
1	Stems	10	11.5 ± 0.7 ^f^	0.0 ^a^	21.6 ± 1.5 ^bd^	0.0 ^a^	24.0 ± 1 ^dkm^	20.0 ± 1 ^cef^
1	Stems	50	19.5 ± 2.1 ^ceij^	0.0 ^a^	22.0 ± 0 ^df^	0.0 ^a^	23.0 ± 0 ^klm^	13.6 ± 0.5 ^bd^
1	Stems	100	09.5 ± 0.7 ^f^	0.0 ^a^	19.6 ± 0.5 ^e^	0.0 ^a^	19.6 ± 0.5 ^ac^	09.6 ± 0.5 ^dgh^
1	Stems	Gentamicin	30.5 ± 0.7 ^ag^	35.0 ± 1.0 ^h^	34.0 ± 0 ^j^	35.0 ± 0 ^f^	33.0 ± 0 ^ij^	27.6 ± 0.7 ^dg^
2	Leaves	10	17.5 ± 0.7 ^cde^	22.0 ±1.0 ^b^	24.3 ± 1.1 ^bc^	14.0 ± 1 ^b^	19.0 ± 1 ^ac^	14.6 ± 0.5 ^bcd^
2	Leaves	50	20.5 ± 2.1 ^cij^	20.7 ± 1.1.0 ^bd^	25.5 ± 0.5 ^c^	11.6 ± 0.5 ^cd^	15.0 ± 1 ^b^	09.6 ± 0.5 ^dgh^
2	Leaves	100	18.5 ± 2.1 ^cdei^	20.0 ± 1.0 ^bd^	19.0 ± 1 ^e^	09.3 ± 0.5 ^e^	0.0 ^f^	0.0 ^a^
2	Leaves	Gentamicin	30.0 ± 1.4 ^ag^	35.0 ± 0.0 ^h^	42.0 ± 0 ^i^	34.6 ± 0.5 ^f^	34.6 ± 0.5 ^j^	31.0 ± 0 ^ij^
2	Stems	10	25.0 ± 1.4 ^hl^	0.0 ^a^	0.0 ^a^	20.0 ± 1 ^h^	18.6 ± 0.5 ^ac^	20.6 ± 1.5 ^ef^
2	Stems	50	21.0 ± 1.4 ^ijm^	0.0 ^a^	0.0 ^a^	17.0 ± 1 ^i^	25.3 ± 1.1 ^dm^	17.0 ± 1.7 ^bce^
2	Stems	100	18.5 ± 0.7 ^cdei^	0.0 ^a^	0.0 ^a^	10.0 ± 0 ^cde^	20.3 ± 1.1 ^cl^	04.6 ± 0.5 ^ah^
2	Stems	Gentamicin	34.0 ± 0.0 ^b^	34.7 ± 0.5 ^h^	37.6 ± 0.5 ^g^	31.0 ± 1 ^j^	35.3 ± 1.1 ^j^	26.3 ± 2.8 ^ik^

Note: Different superscript letters within a column in a time row indicate significant differences among mean observations (*p* < 0.05) for each bacterial strain.

**Table 5:** Table indicating the ZnO NPs from the different parts of the plant and the different precursor concentrations (0.5 M, 1 M, and 2 M) exhibiting the highest antibacterial activity for each bacterial species.

	0.5 M Stems	0.5 M Leaves	1 M Stems	1 M Leaves	2 M Stems	2 M Leaves
*Staphylococcus aureus* ATCC 25923				10 µg/mL		
*Listeria monocytogenes* ATCC 19115						10 µg/mL
*Bacillus cereus* ATCC 11768			10 µg/mL			
*Escherichia coli* ATCC 25922					10 µg/mL	
*Salmonella enteritidis* ATCC 13076		50 µg/mL				
*Klebsiella pneumoniae* ATCC 70603		10 µg/mL				

**Table 6:** ANOVA test results indicate the different levels of significance of the interactions between NP concentration (CC), precursor concentration (CP), and part of the plant (PP) during the synthesis of ZnO NPs from *Nepeta nepetella* subps. *amethystine* against *Staphylococcus aureus* (*S. aureus*), *Listeria monocytogenes* (*L. monocytogenes*), *Bacillus cereus* (*B. cereus*), *Escherichia coli* (*E. coli*), *Salmonella enteritidis* (*S. enteritidis*), and *Klebsiella pneumonia* (*K. pneumonia*).

	*S. aureus*	*L. monocytogenes*	*B. cereus*	*E. coli*	*S. enteritidis*	*K. pneumoniae*
CC	***	***	***	***	***	***
CP	***	***	**	***	***	***
PP	***	***	***	***	***	***
CC:CP	***	***	***	***	***	***
CC:PP	***	***	***	***	***	***
CP:PP	***	***	***	***	***	***
CC:CP:PP	***	***	***	***	***	***

Note: Significance codes: 0, ‘***’ 0.001 and ‘**’ 0.01.

## Data Availability

All data generated or analyzed during this study are included in this published article. Any further specific data analysis can be obtained by making a reasonable request to the corresponding authors.
